# Supplementary material for: High-Throughput Root Imaging Analysis Reveals Wide Variation in Root Morphology of Wild Adzuki bean (Vigna angularis) Accessions
Source: Plants (Basel). 2022 Feb 1;11(3):405. doi: 10.3390/plants11030405 (PMC8840753; doi:10.3390/plants11030405)
Supplement: Supplementary file 1 [file plants-11-00405-s001.zip › plants-1573972-supplementary.pdf]

Table S1. List of adzuki bean accessions used in this experiment.

S. N	IT number	Country of origin	Scientific name
1	236774	Korea	*Vigna angularis* var. *angularis*
2	236775	Korea	*Vigna angularis* var. *angularis*
3	242846	Korea	*Vigna angularis* var. *nipponensis*
4	262413	Korea	*Vigna angularis* var. *nipponensis*
5	262417	Korea	*Vigna angularis* var. *nipponensis*
6	262422	Korea	*Vigna angularis* var. *nipponensis*
7	262423	Korea	*Vigna angularis* var. *nipponensis*
8	262424	Korea	*Vigna angularis* var. *nipponensis*
9	262434	Korea	*Vigna angularis*
10	262441	Korea	*Vigna angularis* var. *nipponensis*
11	262443	Korea	*Vigna angularis*
12	262445	Korea	*Vigna angularis* var. *nipponensis*
13	262446	Korea	*Vigna angularis* var. *nipponensis*
14	262451	Korea	*Vigna angularis* var. *nipponensis*
15	262452	Korea	*Vigna angularis* var. *nipponensis*
16	262454	Korea	*Vigna angularis* var. *nipponensis*
17	262456	Korea	*Vigna angularis* var. *nipponensis*
18	262457	Korea	*Vigna angularis* var. *nipponensis*
19	262464	Korea	*Vigna angularis* var. *nipponensis*
20	262465	Korea	*Vigna angularis* var. *nipponensis*
21	262466	Korea	*Vigna angularis* var. *nipponensis*
22	262469	Korea	*Vigna angularis* var. *nipponensis*
23	262470	Korea	*Vigna angularis* var. *nipponensis*
24	262471	Korea	*Vigna angularis* var. *nipponensis*
25	262472	Korea	*Vigna angularis* var. *nipponensis*
26	262477	Korea	*Vigna angularis* var. *nipponensis*
27	262478	Korea	*Vigna angularis* var. *nipponensis*
28	262479	Korea	*Vigna angularis* var. *nipponensis*
29	262483	Korea	*Vigna angularis* var. *nipponensis*
30	262484	Korea	*Vigna angularis* var. *nipponensis*
31	262485	Korea	*Vigna angularis* var. *nipponensis*
32	262487	Korea	*Vigna angularis* var. *nipponensis*
33	262492	Korea	*Vigna angularis*
34	262493	Korea	*Vigna angularis*
35	262496	Korea	*Vigna angularis* var. *nipponensis*
36	262497	Korea	*Vigna angularis* var. *nipponensis*
37	262498	Korea	*Vigna angularis* var. *nipponensis*
38	262499	Korea	*Vigna angularis* var. *nipponensis*
39	262500	Korea	*Vigna angularis* var. *nipponensis*
40	262501	Korea	*Vigna angularis* var. *nipponensis*
41	262502	Korea	*Vigna angularis* var. *nipponensis*
42	305544	Korea	*Vigna angularis* var. *nipponensis*
43	305585	Korea	*Vigna angularis* var. *nipponensis*
44	305586	Korea	*Vigna angularis* var. *nipponensis*
45	305588	Korea	*Vigna angularis* var. *nipponensis*
46	305589	Korea	*Vigna angularis* var. *nipponensis*

47	305591	Korea	*Vigna angularis* var. *nipponensis*
48	305594	Korea	*Vigna angularis* var. *nipponensis*
49	305595	Korea	*Vigna angularis* var. *nipponensis*
50	305597	Korea	*Vigna angularis* var. *nipponensis*
51	305598	Korea	*Vigna angularis* var. *nipponensis*
52	305599	Korea	*Vigna angularis* var. *nipponensis*
53	305600	Korea	*Vigna angularis* var. *nipponensis*
54	305601	Korea	*Vigna angularis* var. *nipponensis*
55	305602	Korea	*Vigna angularis* var. *nipponensis*
56	305603	Korea	*Vigna angularis* var. *nipponensis*
57	305604	Korea	*Vigna angularis* var. *nipponensis*
58	305605	Korea	*Vigna angularis var. nipponensis*
59	305606	Korea	*Vigna angularis* var. *nipponensis*
60	305607	Korea	*Vigna angularis* var. *nipponensis*
61	305608	Korea	*Vigna angularis* var. *nipponensis*

2
